# Decreasing Antibody Titers and the Slow Decay of Measles Immunity in Mexico’s Current Epidemiological Landscape

**DOI:** 10.3390/vaccines14030234

**Published:** 2026-03-04

**Authors:** José Francisco Muñoz-Valle, Gabriela Macedo-Ojeda, Francisco Javier Turrubiates-Hernández, Jorge Hernández-Bello, Samuel García-Arellano, Cristian Oswaldo Hernández-Ramírez, Christian Johana Baños-Hernández, Pablo Moisés Coronado-Carrillo, Juan Carlos Lona-Reyes, Oliver Viera-Segura

**Affiliations:** 1 Instituto de Investigación en Ciencias Biomédicas, Centro Universitario de Ciencias de la Salud, Universidad de Guadalajara, Guadalajara 44340, Mexico; biologiamolecular@hotmail.com (J.F.M.-V.); gabriela.macedo@cucs.udg.mx (G.M.-O.); francisco.turrubiates@cucs.udg.mx (F.J.T.-H.); jorge89_5@hotmail.com (J.H.-B.); samuel.g.arellano@gmail.com (S.G.-A.); cristian.hernandez3703@alumnos.udg.mx (C.O.H.-R.); johana.banos@academicos.udg.mx (C.J.B.-H.); pablo.coronado.carrillo@gmail.com (P.M.C.-C.); 2 Servicio de Alergia e Inmunología Clínica Pediátrica, Hospital Civil de Guadalajara “Dr. Juan I. Menchaca”, Guadalajara 44340, Mexico; carloslona5@hotmail.com

**Keywords:** measles, vaccine, vaccine-induced immunity

## Abstract

**Background:** Measles remains a global public health threat despite the availability of an effective vaccine and substantial progress toward elimination in many countries. Outbreaks in highly vaccinated settings suggest that waning vaccine-induced immunity, particularly among adults, may create silent susceptibility gaps capable of sustaining viral transmission. **Aim:** To evaluate age- and cohort-specific patterns of measles-specific antibody levels in Mexican adults and to examine evidence suggesting lower vaccine-induced antibody levels in younger vaccinated cohorts. **Methods:** A cross-sectional seroepidemiological study was conducted among 302 voluntary blood donors aged 18–70 years. Demographic, clinical, and vaccination data were collected through structured interviews. Serum anti-measles-virus (MV) IgG levels were quantified using a commercial ELISA. Antibody concentrations were analyzed according to age, sex, vaccination history, self-reported measles infection, and historical vaccination strategies. Multivariate linear models were applied to identify factors associated with IgG levels. **Results:** Anti-MV IgG seropositivity (>200 mIU/mL) was 67.2%, with a geometric mean concentration (GMC) of 270.43 mIU/mL. A positive correlation was observed between age and antibody levels (r_s_ = 0.161, *p* = 0.005). Individuals born before the introduction of the measles vaccine (pre-1970) had significantly higher GMCs (1096.63 mIU/mL) than younger cohorts. A history of natural infection tended to be associated with higher antibody levels (GMC: 428.38 vs. 257.24 mIU/mL; *p* = 0.051). In multivariate analysis, historical vaccination strategy emerged as the primary factor associated with antibody levels, whereas age alone was not significant. **Conclusions:** Cohort-specific differences in measles IgG levels suggest generational patterns of immunity and are consistent with diminished vaccine-induced antibody levels in younger adults in the absence of natural boosting. These findings highlight the importance of ongoing serological surveillance in post-elimination settings and underscore the need for targeted public health interventions.

## 1. Introduction

Measles is an acute, highly contagious viral disease exclusive to humans that remains a significant public health threat. The disease is caused by *Measles morbillivirus* (MV), an enveloped, single-stranded RNA virus belonging to the *Paramyxoviridae* family. Its remarkable transmissibility is reflected in a basic reproduction number (R_0_), ranging from 12 to 18 [1,2,3]. The measles virus exhibits strict host selectivity, largely mediated by its interaction with the signaling lymphocytic activation molecule (SLAMF1/CD150) expressed on immune cells and nectin-4 on epithelial cells, which together enable systemic dissemination and respiratory transmission [4]. Although infection can occur in non-human primates [5], sustained transmission in wild animal populations has not been demonstrated, supporting humans as the sole natural reservoir and reinforcing the feasibility of measles elimination through vaccination strategies [3].

Prior to the widespread adoption of vaccination programs, this high transmissibility resulted in an estimated 2.6 million measles-related deaths annually worldwide [1,2,3]. The introduction of a safe and effective measles vaccine, first licensed in 1963, represented a major public health milestone [6]. The Schwarz measles vaccine became available in the 1970s and was subsequently replaced by the Edmonston-Zagreb strain in 1978. A two-dose childhood immunization schedule was introduced in 1991, and in 1998, the combined measles-mumps-rubella (MMR) vaccine was incorporated into national immunization programs [7,8]. The impact of these vaccination strategies has been substantial; between 2000 and 2022, measles vaccination is estimated to have prevented approximately 57 million deaths globally [7].

Despite these achievements, recent epidemiological trends are concerning. In 2022, global measles-related deaths increased to an estimated 107,482, with the majority occurring among unvaccinated or under-vaccinated children under the age of five. While vaccination programs have dramatically reduced measles mortality, they have also contributed to an evolving epidemiological landscape in which waning vaccine-induced immunity—rather than primary vaccine failure—may represent the next major challenge to measles elimination [9].

The recent resurgence of measles threatens global elimination goals and underscores persistent immunity gaps. Global coverage with the first dose of the measles-containing vaccine declined from 86% in 2019 to 81% in 2021, largely due to pandemic-related disruptions to routine immunization services and increasing vaccine hesitancy [9]. These trends have already translated into outbreaks in settings previously considered protected. In February 2024, the World Health Organization warned that more than half of the world’s countries could be at risk of measles outbreaks [9]. This global pattern is reflected at the national level; Mexico experienced a measles outbreak in 2020 that primarily affected adults in urban areas, and international health authorities reported confirmed cases in the country in early 2025. These events suggest that reliance on overall vaccination coverage alone may be insufficient, underscoring the need to examine the long-term durability of both humoral and cell-mediated immunity among previously vaccinated individuals [7,10,11].

Natural measles infection confers robust, long-lasting immunity, characterized by sustained neutralizing IgG antibody levels. In contrast, the immune response elicited by the live-attenuated measles vaccine is quantitatively lower and tends to wane over time. Vaccine-induced antibody titers are generally lower and less persistent than those generated following natural infection [11,12]. This decline may result in secondary vaccine failure, defined as the loss of protective immunity over time despite an initially adequate immune response. It is estimated that secondary vaccine failure occurs in approximately 5% of individuals 10–15 years after immunization [13,14].

In Mexico, a national serological survey reported an overall measles seroprevalence of 82.4% as measured by the plaque-reduction neutralization test (PRNT). Notably, the lowest seroprevalence was observed among individuals aged 20–29 years, at 63.6%. This represents a substantial decline compared with a 2012 national survey, which reported a seroprevalence of 95% across all age cohorts, highlighting a marked reduction in immunity in specific cohorts over the past decade [7]. Similar patterns have been reported internationally. In Brazil, Castiñeiras et al. found that the time elapsed since the last measles-mumps-rubella (MMR) vaccine dose was negatively associated with seropositivity, indicating a progressive decline in antibody levels with increasing time since vaccination [15]. Likewise, a longitudinal study in China estimated, based on observed antibody decay rates, that measles antibody concentrations could fall below protective thresholds at a median age of 14.3 years [16].

Together, this evidence from diverse populations in Latin America and Asia reveals a consistent and concerning picture: vaccine-induced antibody levels are declining over time, with young adults representing the most vulnerable group. The accumulation of susceptible individuals within previously vaccinated cohorts, particularly among young adults, represents a growing threat to measles elimination efforts. This silent erosion of herd immunity underscores the need for precise quantification of antibody levels in adult populations to identify immunity gaps and guide public health strategies. Therefore, the objective of the present study was to evaluate age- and cohort-specific patterns of measles-specific antibody levels in Mexican adults and to examine evidence consistent with diminishing vaccine-induced immunity in younger vaccinated cohorts.

## 2. Materials and Methods

### 2.1. Study Design and Population

A cross-sectional study was conducted to assess the seroprevalence of anti-measles virus (MV) antibodies among blood donors attending the *Instituto de Investigación en Ciencias Biomédicas (IICB)* at the *Universidad de Guadalajara*. A total of 302 participants were included. Eligible participants were adults aged 18–70 who voluntarily agreed to participate and provided written informed consent. Data on documented doses of the measles-mumps-rubella (MMR) or measles-mumps-rubella-varicella (MMRV) vaccines, as well as self-reported history of natural measles infection, were collected when available. Each participant completed a structured interview to obtain demographic information and data related to MV exposure and vaccination, occupation, comorbidities, and vaccination history.

### 2.2. Sample Collection and Serological Analysis

Blood samples were obtained from all enrolled participants, and serum was separated and stored at −20 °C until analysis. Anti-MV IgG concentrations were measured using a commercial enzyme-linked immunosorbent assay (ELISA) kit (SERION ELISA classic Measles Virus IgG; Institut Virion/Serion GmbH, Würzburg, Germany).

According to the manufacturer’s recommendations, antibody concentrations were classified as negative (<150 mIU/mL) or positive (>200 mIU/mL). Values within the equivocal range correspond to the assay cut-off and do not indicate clinical protection against measles infection. All assays were performed according to the manufacturer’s instructions. Optical density was measured at 405 nm using a Thermo Scientific (Waltham, MA, USA) Multiskan GO microplate spectrophotometer.

### 2.3. Statistical Analysis

Qualitative variables were summarized as frequencies and percentages, while quantitative variables were described using medians, interquartile ranges (IQRs), and geometric means, as appropriate. One-way analysis of variance (ANOVA) was used to compare mean antibody concentrations between groups, followed by Tukey’s post hoc test for multiple comparisons. Multivariate linear regression models were applied to identify factors independently associated with anti-MV IgG concentrations. Correlation analyses were also performed to assess associations between antibody levels and explanatory variables. Statistical analyses were conducted using SPSS version 26 (IBM Corp., Armonk, NY, USA) and GraphPad Prism version 8 (GraphPad Software, San Diego, CA, USA). A two-tailed *p*-value < 0.05 was considered statistically significant.

## 3. Results

### 3.1. Sociodemographic and Clinical Characteristics

A total of 302 participants were included in the study. The median age was 26 years (IQR: 19–35). Female participants accounted for 57.6% of the sample, and most individuals were born in the Central region of Mexico (84.8%). The majority of participants reported a high level of education, with 61.3% holding a bachelor’s degree or a postgraduate qualification.

Comorbidities were reported by 14.2% of the study population (*n* = 43), with arterial hypertension being the most frequently reported condition. Regarding measles vaccination status, 31.5% reported being vaccinated but could not recall the number of doses received, while 30.8% reported receiving two doses. Additionally, 14 participants reported a history of natural measles infection. Detailed sociodemographic and clinical characteristics are presented in Table 1.

### 3.2. Anti-Measles IgG Levels According to Clinical History and Recent Infection

To evaluate the immunological status of the study population, anti-measles IgG concentrations were quantified. Overall, 67.2% of participants were seropositive (>200 mIU/mL), with a geometric mean concentration (GMC) of 270.43 mIU/mL (95% CI: 244.69–298.87; Table 1).

**Figure 1:**
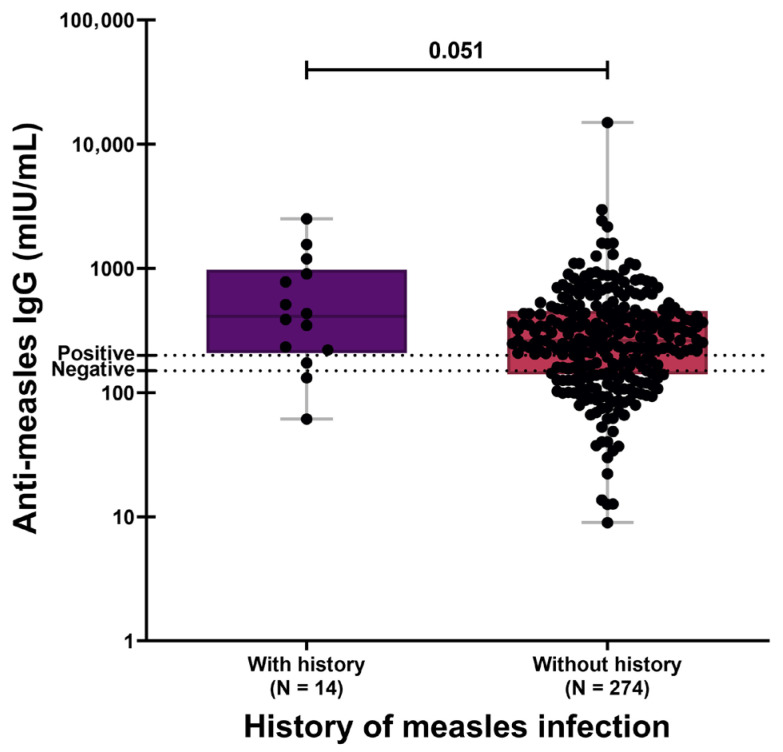
Anti-measles IgG concentrations to history of measles infection. Individuals reporting prior measles showed a trend toward higher antibody levels. The *y*-axis is displayed on a logarithmic (log_10_) scale. The dotted horizontal lines denote the manufacturer’s seropositivity threshold.

Participants reporting a history of natural measles infection exhibited higher antibody concentrations (GMC: 428.38 mIU/mL; 95% CI: 237.46–772.78) compared with those without prior infection (GMC: 257.24 mIU/mL; 95% CI: 230.44–290.03), although this difference did not reach conventional statistical significance (Figure 1; *p* = 0.051). This trend persisted when the analysis was restricted to seropositive individuals only (Supplementary Table S1). In contrast, the presence of a recent nonspecific infection within the previous 3 months was not associated with differences in anti-measles IgG concentrations.

**Figure 2:**
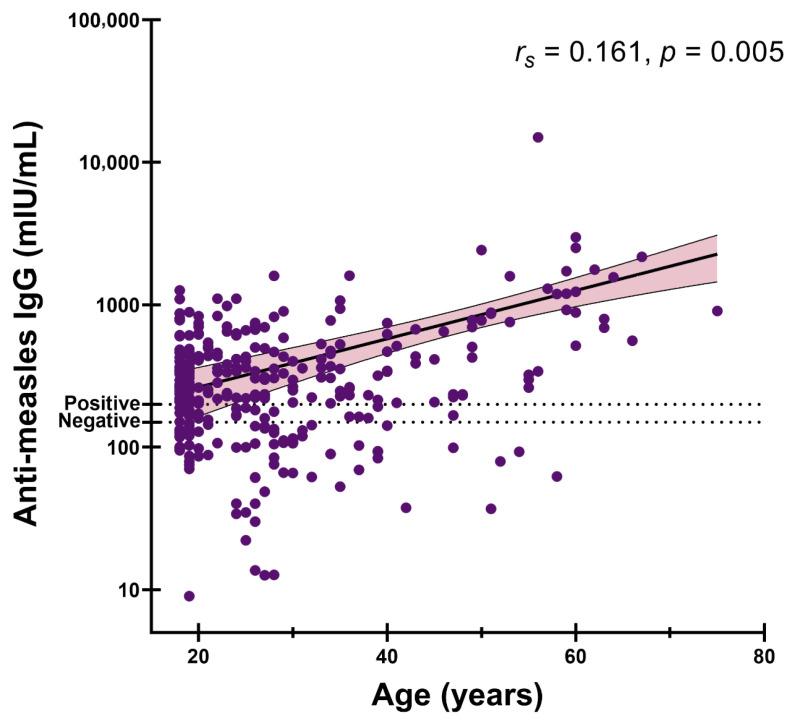
Correlation between age and anti-measles IgG concentrations in the study population. Anti-measles IgG levels are plotted against age for all participants. The *y*-axis is presented on a logarithmic (log_10_) scale. The solid line represents the fitted regression line, and the shaded area indicates the 95% confidence interval. The dotted horizontal lines denote the manufacturer’s seropositivity threshold.

To explore age-related patterns in immunity, we first evaluated the correlation between anti-measles IgG levels and age in the overall cohort. A weak but statistically significant positive correlation was identified (Spearman’s *r_s_* = 0.161, *p* = 0.005; Figure 2). When the analysis was restricted to seropositive individuals (>200 mIU/mL), the association persisted and strengthened (*r_s_* = 0.280, *p* < 0.001; Supplementary Figure S1).

Furthermore, analysis of anti-measles IgG concentrations by sex revealed no statistically significant differences between females (GMC: 287.15 mIU/mL; 95% CI: 244.69–333.62) and males (GMC: 249.64 mIU/mL; 95% CI: 214.86–287.15; *p* = 0.219). Results of the sex-stratified analysis restricted to seropositive individuals are provided in Supplementary Table S2.

### 3.3. Serology According to Historical Vaccination Schemes

**Figure 3:**
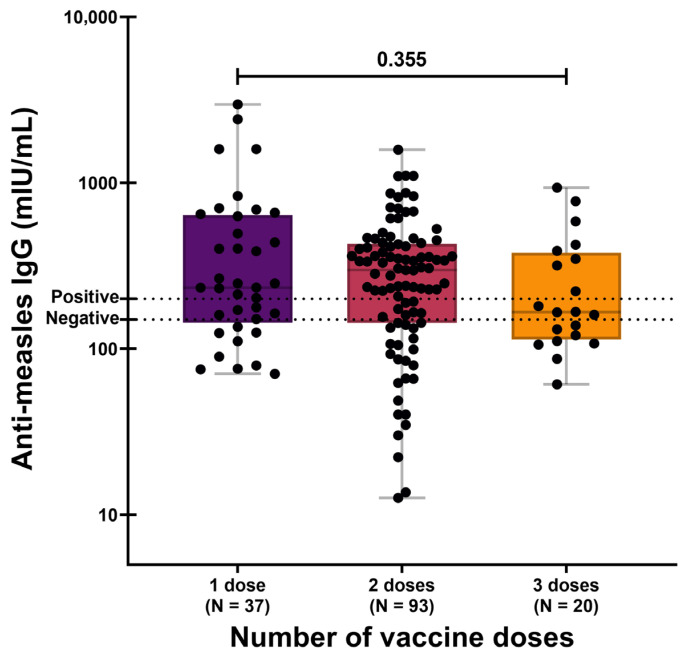
Anti-measles IgG concentrations by number of reported vaccine doses. Data are presented on a logarithmic (log_10_) scale. One-way analysis of variance (ANOVA) was performed using log-transformed antibody concentrations, followed by Tukey’s post hoc test. The dotted horizontal lines denote the manufacturer’s seropositivity threshold.

As described above, three distinct measles vaccination strategies have been implemented in Mexico across different decades, each characterized by a different number of administered doses. When anti-measles IgG concentrations were analyzed according to the reported number of vaccine doses, no statistically significant differences were observed. The geometric mean concentrations (GMCs) were 292.95 mIU/mL (95% CI: 210.61–407.48) among individuals reporting one dose, 237.46 mIU/mL (95% CI: 194.42–290.03) among those reporting two doses, and 206.44 mIU/mL (95% CI: 145.47–292.95) among those reporting three doses (*p* = 0.355; Figure 3).

These findings remained consistent when analyses were restricted to seropositive individuals only (Supplementary Figure S2 and Table S3).

**Figure 4:**
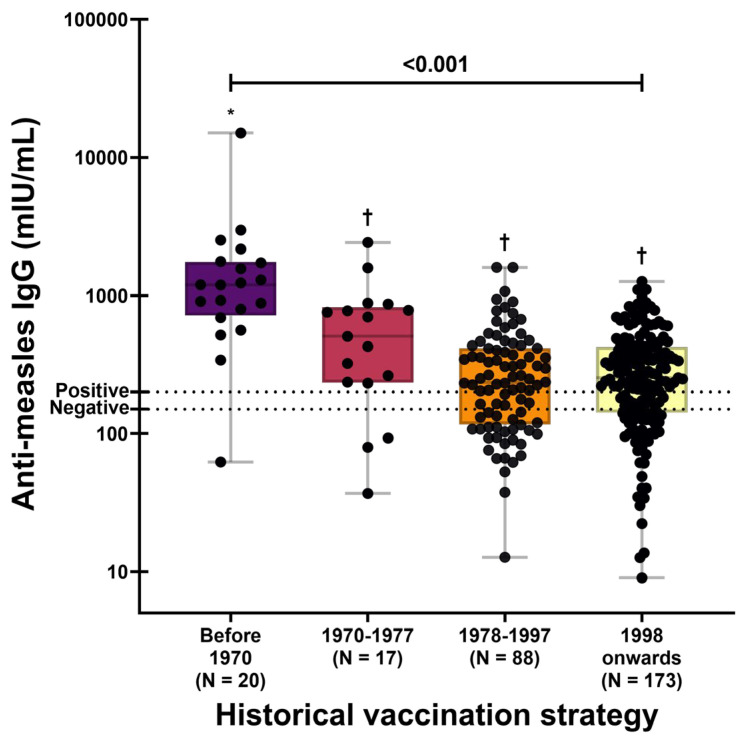
Anti-measles IgG concentrations according to historical measles vaccination strategies in Mexico. Data are presented on a logarithmic scale (log_10_). A one-way ANOVA was performed on log-transformed antibody concentrations, followed by Tukey’s post hoc test. Different symbols above box plots indicate statistically significant differences between groups. The dotted horizontal lines denote the manufacturer’s seropositivity threshold.

When participants were subsequently stratified according to the historical measles vaccination strategy in Mexico, significant differences in anti-measles IgG concentrations were observed (*p* < 0.001; Figure 4). Individuals born before 1970 (pre-vaccination era) exhibited the highest antibody levels (GMC: 1096.63 mIU/mL; 95% CI: 671.83–1790.05). In contrast, IgG concentrations among younger birth cohorts were comparatively homogeneous, with GMCs of 403.43 mIU/mL for those born between 1970 and 1977 (95% CI: 237.46–685.4), 230.44 mIU/mL for those born between 1978 and 1997 (95% CI: 192.48–278.66), and 237.46 mIU/mL for those born in 1998 or later (95% CI: 208.51–270.43).

In addition, a significant negative correlation was observed between progression of vaccination strategies over time and anti-measles IgG concentrations (Spearman’s *r_s_* = −0.205, *p* < 0.001), indicating lower antibody levels in younger birth cohorts. This association was confirmed in analyses restricted to seropositive participants (Supplementary Figure S3).

### 3.4. Factors Associated with Anti-Measles IgG Levels

Multiple factors may influence antibody production against infectious pathogens. To identify variables associated with anti-measles IgG concentrations, a generalized linear model adjusted for age, sex, history of measles infection, and vaccination status was fitted. In this model, age was significantly associated with higher anti-measles IgG concentrations (*β* = 0.021; 95% CI: 0.013–0.030; *p* < 0.001), corresponding to an estimated 2.1% increase in antibody levels per additional year of age (Table 2).

Nevertheless, after including the historical vaccination strategy in a second model, the association with age was no longer statistically significant (*β* = −0.009; *p* = 0.521). In contrast, vaccination strategy showed a strong association with IgG levels (*p* < 0.001). Participants born before 1970 exhibited significantly higher antibody concentrations (*β* = 1.799; 95% CI: 0.692–2.906) compared with those born in 1998 or later, corresponding to a 6.04-fold increase in geometric mean titers. This model explained 13.5% of the variance in anti-measles IgG levels (Table 3). The loss of statistical significance for age in the adjusted model suggests that the initial age association was largely driven by cohort-specific patterns rather than chronological aging itself.

## 4. Discussion

Measles remains a significant global public health challenge due to its extreme transmissibility and its capacity to exploit even small gaps in population immunity, making it one of the first vaccine-preventable diseases to re-emerge when immunization programs are disrupted [1]. Despite the availability of safe and highly effective vaccines, measles resurgence has been reported in recent years, driven by interruptions to routine immunization services during the COVID-19 pandemic, the accumulation of susceptible cohorts, and increasing vaccine hesitancy fueled by misinformation [17].

In Mexico, these global trends have translated into recent outbreaks, highlighting the persistence of susceptibility pockets across age groups and regions and underscoring the fragility of measles elimination achievements [7,8,18]. In this context, sustaining high vaccination coverage—complemented by seroepidemiological surveillance and systematic assessments of population immunity—is essential to prevent the re-establishment of endemic transmission and to inform more effective measles control strategies.

Mexico has a long history of measles vaccination programs. The initial measles vaccine introduced in 1970 was based on the Schwarz strain, followed by the Edmonston-Zagreb strain in 1978; both were administered as monovalent vaccines and were subsequently replaced in 1998 by the combined MMR vaccine. While the present study focuses on anti-measles IgG, it is noteworthy that a concomitant waning of vaccine-induced antibody titers against the mumps and rubella components of the MMR vaccine has also been reported in young adults, a phenomenon that contributes to the re-emergence of these diseases in highly vaccinated populations [19,20,21,22]. Following the widespread implementation of the vaccination programs in Mexico, indigenous measles transmission was considered interrupted in the country [8,10]. However, vaccination coverage has declined in recent years, decreasing from 75.03% in 2012 to 71.3% in 2023 among children under five years of age [10]. Coverage among individuals aged 10–19 years has also declined, reaching only 61.8%, while coverage among young adults and older adults remains particularly low at 47.67% [23,24]. Insufficient vaccination coverage—especially incomplete two-dose schedules—has resulted in only 32.7% coverage among children under three years of age [8], increasing the risk of inadequate measles protection and subsequent outbreaks.

In the present study, 67.2% of participants were seropositive for anti-measles antibodies. This estimate exceeds the 47.67% reported by Mongua-Rodríguez et al. for adults and approximates the 61.8% observed among individuals under 18 years of age in the same report [23]. Notably, antibody concentrations differed according to historical vaccination strategy, with cohorts including older adults exhibiting higher titers than those including younger individuals. This cohort-related age pattern is consistent with previous studies reporting higher anti-measles antibody levels in older populations [25,26,27] and has been attributed to repeated exposure to circulating wild-type measles virus, which may boost antibody titers through subclinical reinfection [27].

In Mexico, this interpretation is epidemiologically plausible. Adults born before measles interruption of endemic transmission were exposed to circulating wild-type virus throughout the 1970s and 1980s, and many experienced the large national epidemic of 1989–1990. Such exposure likely led to natural infection and subsequent immune boosting, resulting in higher and more durable antibody titers in older adults. In contrast, younger adults vaccinated in childhood relied exclusively on vaccine-induced immunity without natural boosting, which may help explain their lower antibody levels and increased susceptibility.

Together, these findings support a cohort effect in which older adults retain immunity derived from natural infection, whereas younger cohorts rely solely on vaccine-induced immunity. As endemic circulation ceases, these differences acquire population-level relevance. Declining antibody levels, combined with decreasing vaccination coverage, may progressively erode herd protection as older immune cohorts are replaced by younger adults with lower seropositivity, as suggested by Williamson et al. (2024) [25]. Similar declines in measles antibodies have been documented in children, in whom seroprotection decreases four to seven years after vaccination [28], indicating that waning immunity is most pronounced shortly after primary immunization, with partial recovery observed later in adulthood. This phenomenon reflects a broader paradox of post-elimination settings: interruption of viral circulation eliminates natural immune boosting while allowing vaccinated cohorts with waning immunity to accumulate. Moreover, reduced circulation may foster a false sense of safety and diminish perceived need for vaccination, thereby facilitating outbreaks [29].

Before the first introduction of measles vaccination in Mexico in 1970, immunity was acquired exclusively through natural infection. The initial Schwarz strain vaccine was administered as a single dose and may have required booster doses to ensure durable protection. In 1978, this strain was replaced by Edmonston-Zagreb, which demonstrated improved immunogenicity [28], and both were later superseded by the trivalent MMR vaccine containing the attenuated Edmonston strain [30]. The higher antibody titers observed in the pre-vaccination cohort strongly suggest that natural infection was the principal driver of long-lasting immunity. It is well established that immunity acquired through natural infection confers more robust and lifelong protection against secondary measles infection [11,12], a conclusion reinforced by meta-analysis evidence indicating increased susceptibility among healthcare professionals vaccinated exclusively in the post-vaccination era [31]. This pattern aligns with our findings, in which participants reporting prior measles infection exhibited higher antibody levels, although the difference did not reach statistical significance. Importantly, despite these differences, vaccination remains an effective and indispensable strategy for preventing measles disease.

Consistent with this interpretation, participants born before 1970 exhibited markedly higher antibody levels than those born in the post-vaccination era, supporting the role of natural infection followed by immune boosting. Collectively, these data reinforce the notion that observed differences in antibody levels primarily reflect historical exposure and vaccination strategies rather than a biological effect of aging on humoral immunity.

Furthermore, an age-related cohort effect has also been observed in European populations, with an increasing prevalence of negative serology in the youngest generations [32,33,34]. Similar patterns have been reported in Asia [16,35]. In the Americas, a recent study conducted in the United States reported high two-dose serological coverage of 87.8% [36], contrasting with the Latin America scenario, where seroprevalence was 75% in a cohort of children in Brazil [37], and 78.4% in Colombia, where younger populations also showed lower antibody levels [38]. In Argentina, this age-cohort difference was not observed [39], likely due to the restricted age range of the study population (7–19 years). Together, these studies indicate that cohort-age effects are not unique to Mexico but are observed globally.

Sex-related differences in humoral responses have also been reported, with some studies suggesting greater antibody persistence among females [12,40]. In the present cohort, however, no significant sex-based differences were observed in either antibody concentrations or seropositivity rates. This finding contrasts with evidence from a recent meta-analysis identifying sex as a determinant of measles antibody levels [31]. One possible explanation is that age-related effects may have outweighed sex-related differences in this adult population, attenuating detectable sex-based variation.

It is also important to acknowledge that seropositivity thresholds do not represent absolute correlates of protection. Functional immunity to measles involves not only antibody quantity but also neutralizing capacity, antibody avidity, and cellular immune responses. Indeed, outbreaks have been documented in highly vaccinated populations among seropositive individuals, indicating that protection may be context-dependent and influenced by exposure intensity and host factors [19,41,42].

This study has several limitations. The study population consisted of voluntary blood donors, who may differ from the general population in health status and healthcare access, thereby limiting generalizability. Therefore, the observed seroprevalence of 67.2% should not be interpreted as nationally representative of the Mexican adult population. Moreover, the cross-sectional design precludes direct longitudinal assessment of antibody waning. Vaccination history and prior measles infection were self-reported, introducing potential recall bias. Additionally, only IgG concentrations were measured; neutralizing activity, antibody avidity, and cellular immunity were not assessed. Finally, participants were recruited from a single Mexican state, and regional heterogeneity in vaccination coverage and exposure history may influence antibody profiles. However, internal comparisons across birth cohorts remain informative for understanding generational patterns of measles immunity, with important implications for measles incidence reduction efforts and outbreak preparedness.

## 5. Conclusions

In summary, this study provides seroepidemiological evidence that younger adults have lower measles-specific antibody titers than older cohorts, consistent with declining vaccine-induced antibody levels in the absence of natural boosting. Although the clinical and epidemiological implications of these findings require further investigation, young adults may represent a population in which susceptibility to measles progressively accumulates over time. Continued serological surveillance, together with sustained vaccination efforts, is therefore essential to support measles incidence reduction and prevent future outbreaks in Mexico.

## Figures and Tables

**Table 1:** Sociodemographic and clinical characteristics of the study population.

Variable	Total (*N* = 302)
Age (year) ^a^	26 (19–35)
Sex, *n* (%)	
Female	174 (57.6)
Male	128 (42.4)
Educational level, *n* (%)	
Basic	3 (1)
High School	114 (37.7)
Bachelor’s degree	112 (37.1)
Postgraduate degree	73 (24.2)
Region of birth in Mexico, *n* (%)	
North	18 (6)
Center	256 (84.8)
South-central	13 (4.3)
South-Southeast	15 (5)
Comorbidities, *n* (%)	
None	259 (85.7)
Arterial hypertension	11 (3.6)
Diabetes mellitus	5 (1.7)
Asthma	6 (2)
Other	21 (7)
Corticosteroid treatment, *n* (%)	10 (3.3)
Recent nonspecific infection (<3 months), *n* (%) ^b^	68 (22.5)
History of measles vaccination, *n* (%)	
Vaccinated, number of doses unknown	95 (31.4)
One dose	37 (12.3)
Two doses	93 (30.8)
Three doses	20 (6.6)
Unknown	57 (18.9)
History of measles infection, *n* (%)	
Yes	14 (4.6)
No	274 (90.8)
Unknown	14 (4.6)
Anti-measles IgG (mIU/mL) ^c^	270.43 (95% CI: 244.69–298.87)
Anti-measles IgG interpretation, *n* (%)	
Negative (<150 mIU/mL)	78 (25.8)
Indeterminate (150–200 mIU/mL)	21 (7)
Positive (>200 mIU/mL)	203 (67.2)

^a^ Data are presented as median (Q1–Q3); ^b^ Self-reported infectious processes (e.g., respiratory, gastrointestinal, etc.) occurring within the 3 months prior to sample collection; ^c^ Data are presented as geometric mean (95% confidence interval).

**Table 2:** Univariate linear regression analysis of factors associated with anti-measles IgG levels.

Variable	Coefficient (*β*)	CI 95%	*p* Value
Age (year)	0.021	0.013–0.030	**<0.001**
Sex			0.614
Female (reference)	-	-	
Male	−0.056	−0.277–0.164	
History of measles infection			0.204
No history (reference)	-	-	
History of infection	0.339	−0.185–0.863	
History of measles vaccination			0.438
No history (reference)	-	-	
One dose	0.089	−0.247–0.425	
Two doses	−0.117	−0.363–0.129	
Three doses	−0.264	−0.702–0.174	

Adjusted *R*^2^ = 0.078. The dependent variable was anti-measles IgG concentration (log-transformed). CI: confidence interval. Bold values indicate statistical significant associations (*p* < 0.05).

**Table 3:** Multivariate linear regression model including historical vaccination strategy.

Variable	Coefficient (*β*)	95% CI	*p* Value
Historical vaccination strategy			**<0.001**
1998 onward (reference)	-	-	
1978–1997	0.111	−0.314–0.537	
1970–1977	0.801	−0.101–1.703	
Before 1970	1.799	0.692–2.906	
Age (years)	−0.009	−0.035–0.018	0.521
Sex			0.820
Female (reference)	-	-	
Male	−0.025	−0.240–0.190	
History of measles infection			0.360
No history (reference)	-	-	
History of infection	0.240	−0.275–0.754	
History of measles vaccination			0.709
Unknown/cannot recall (reference)	-	-	
One dose	0.136	−0.191–0.462	
Two doses	−0.021	−0.263–0.221	
Three doses	−0.144	−0.574–0.286	

Adjusted *R*^2^ = 0.135. The dependent variable was anti-measles IgG concentration (log-transformed). CI: confidence interval. Bold values indicate statistical significant associations (*p* < 0.05).

## Data Availability

The data supporting the findings of this study are available from the corresponding author (O.V.-S.) upon reasonable request.

## Supplementary Materials

The following supporting information can be downloaded at: https://www.mdpi.com/article/10.3390/vaccines14030234/s1, Table S1: Anti-measles IgG levels in seropositive individuals (>200 mIU/mL) according to history of measles infection; Figure S1: Correlation between age and anti-measles IgG levels. Anti-measles IgG concentrations plotted against age among seropositive participants (>200 mIU/mL). The *y*-axis is presented on a logarithmic (log^10^) scale; Table S2: Anti-measles IgG levels in seropositive individuals (>200 mIU/mL) according to sex; Figure S2: Anti-measles IgG levels in seropositive individuals (>200 mIU/mL) by number of vaccine doses. Data are presented on a logarithmic (log_10_) scale. Group comparisons were performed using one-way ANOVA on log-transformed antibody concentrations, followed by Tukey’s post hoc test; Table S3: Anti-measles IgG levels in seropositive individuals (>200 mIU/mL) by number of vaccine doses; Figure S3: Anti-measles IgG levels in seropositive individuals (>200 mIU/mL) according to historical measles vaccination strategies in Mexico. Data are presented on a logarithmic (log_10_) scale. Group comparisons were performed using one-way ANOVA on log-transformed antibody concentrations, followed by Tukey’s post hoc test. Different symbols above box plots indicate statistically significant differences between groups.

## Abbreviations

The following abbreviations are used in this manuscript:

MV	Measles *morbillivirus*
MMR	Measles, mumps, and rubella vaccine
MMRV	Measles, mumps, rubella, and varicella vaccine
IgG	Immunoglobulin G
IQR	Interquartile range
GMC	Geometric mean concentration
CI	Confidence interval
ELISA	Enzyme-linked immunosorbent assay
PRNT	Plaque-reduction neutralization test
WHO	World Health Organization
R_0_	Basic reproduction number
ANOVA	Analysis of variance
IICB	Instituto de Investigación en Ciencias Biomédicas
COVID-19	Coronavirus disease 2019
