# Supplementary material for: Decreasing Antibody Titers and the Slow Decay of Measles Immunity in Mexico’s Current Epidemiological Landscape
Source: Vaccines (Basel). 2026 Mar 4;14(3):234. doi: 10.3390/vaccines14030234 (PMC13030418; doi:10.3390/vaccines14030234)
Supplement: Supplementary file 1 [file vaccines-14-00234-s001.zip › vaccines-4146984-supplementary.pdf]

Supplementary Materials

Supplementary Table S1. Anti-measles IgG levels in seropositive individuals (>200 mIU/mL) according to history of measles infection.

Variable	With a history of measles infection (N = 11)	Without a history of measles infection (N = 181)	*p* value
**Anti-measles IgG (mIU/mL)[a]**	620.17 (365.04-1043.15)	428.38 (395.44-468.72)	0.060

[a] Data are presented as geometric mean (95% confidence interval). Comparisons were performed using Student's *t*-test for independent samples on log-transformed data.

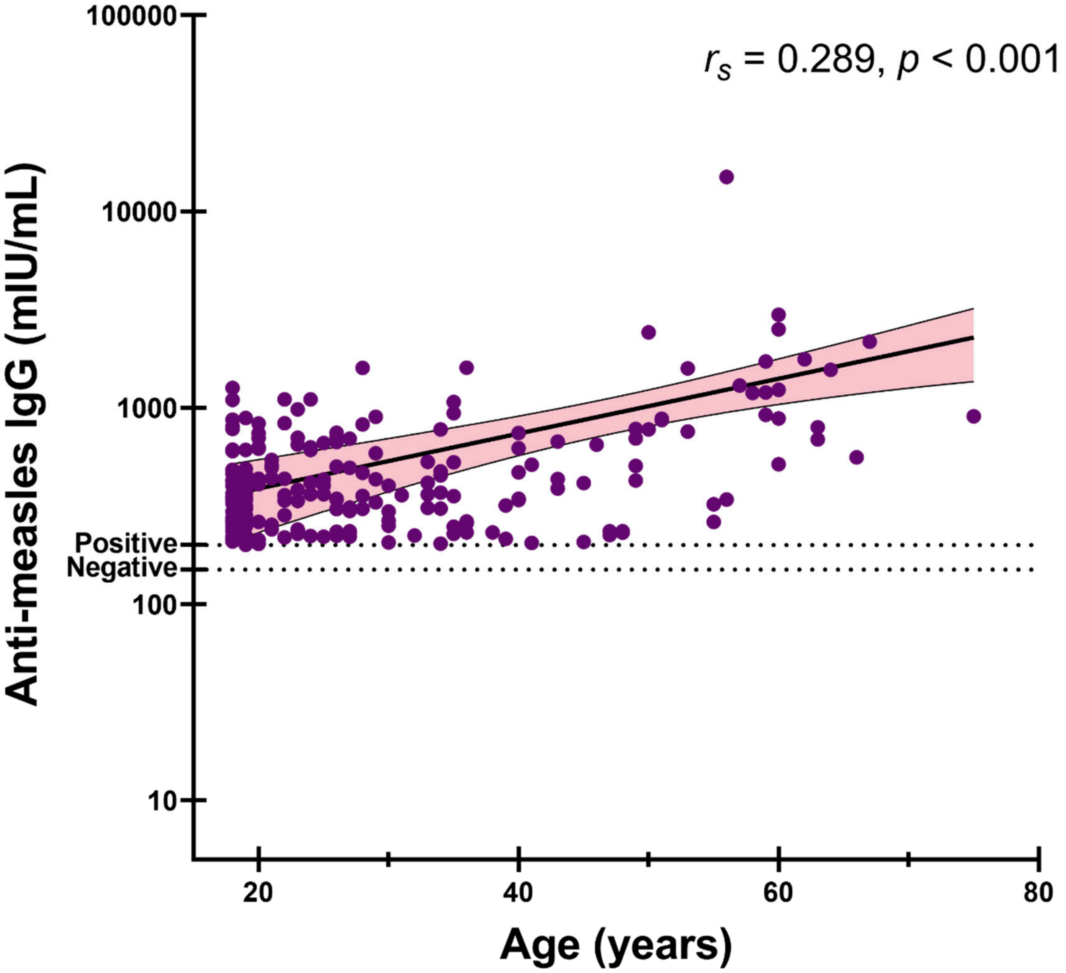

Supplementary Figure S1. Correlation between age and anti-measles IgG levels. Anti-measles IgG concentrations plotted against age among seropositive participants (>200 mIU/mL). The y-axis is presented on a logarithmic (log_{10}) scale. The solid line represents the fitted regression line, and the shaded area indicates the 95% confidence interval. The dotted horizontal lines denote the manufacturer's seropositivity threshold.

**Supplementary Table S2.** Anti-measles IgG levels in seropositive individuals (>200 mIU/mL) according to sex.			
**Variable**	**Females (N = 122)**	**Males (N = 81)**	***p* value**
**Anti-measles IgG (mIU/mL)[a]**	473.43 (415.72-539.15)	419.89 (372.41-468.72)	0.149
[a] Data are presented as geometric mean (95% confidence interval). Comparisons were performed using Student's *t*-test for independent samples on log-transformed data.			

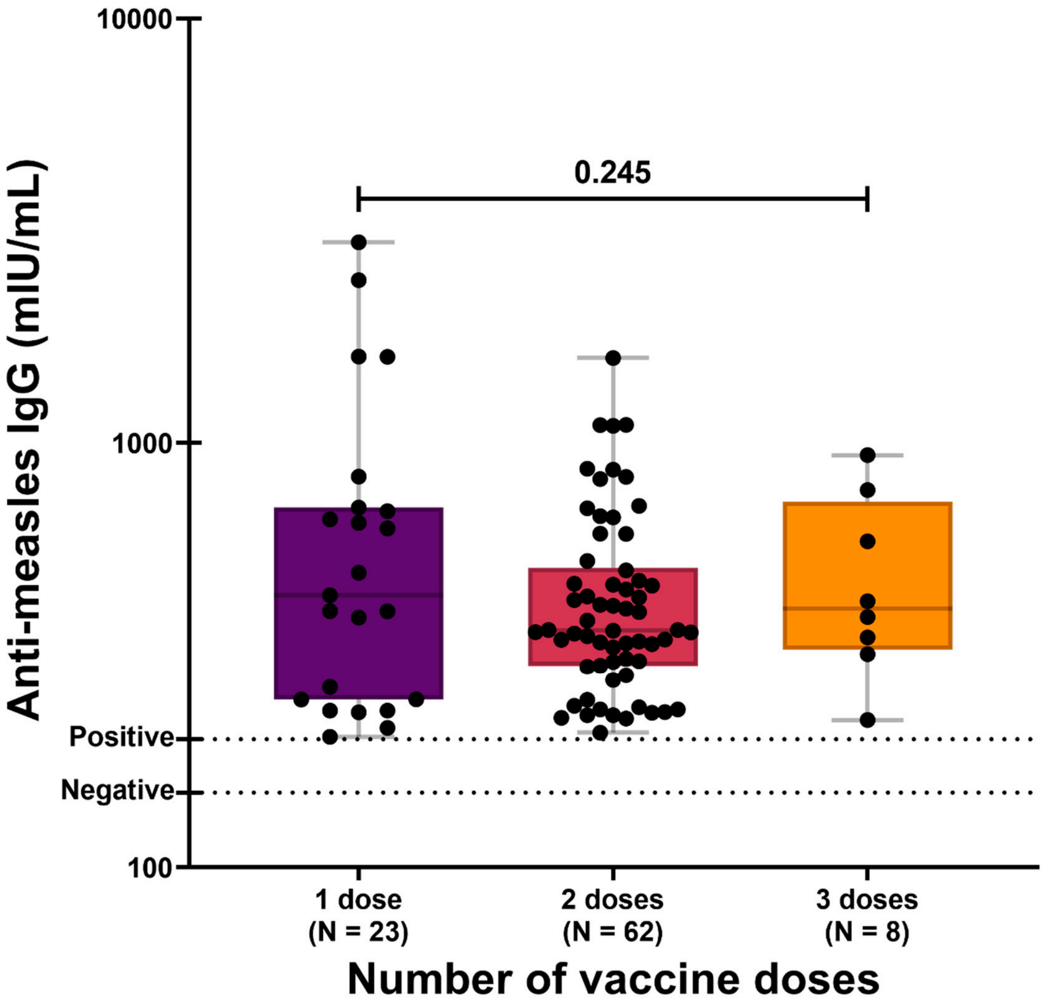

Supplementary Figure S2. Anti-measles IgG levels in seropositive individuals (>200 mIU/mL) by number of vaccine doses. Data are presented on a logarithmic (log_{10}) scale. Group comparisons were

performed using one-way ANOVA on log-transformed antibody concentrations, followed by Tukey's post hoc test. The dotted horizontal lines denote the manufacturer's seropositivity threshold

Supplementary Table S3. Anti-measles IgG levels in seropositive individuals (>200 mIU/mL) by number of vaccine doses.

Variable	1 dose (N = 23)	2 doses (N = 62)	3 doses (N = 8)	*p* value
**Anti-measles IgG (mIU/mL)**[a]	518.01 (365.04 – 727.78)	407.48 (361.41 – 459.44)	450.34 (301.87 – 671.83)	0.245

[a] Data are presented as geometric mean (95% confidence interval). Comparisons were performed using one-way ANOVA on log-transformed data, followed by Tukey's post hoc test.

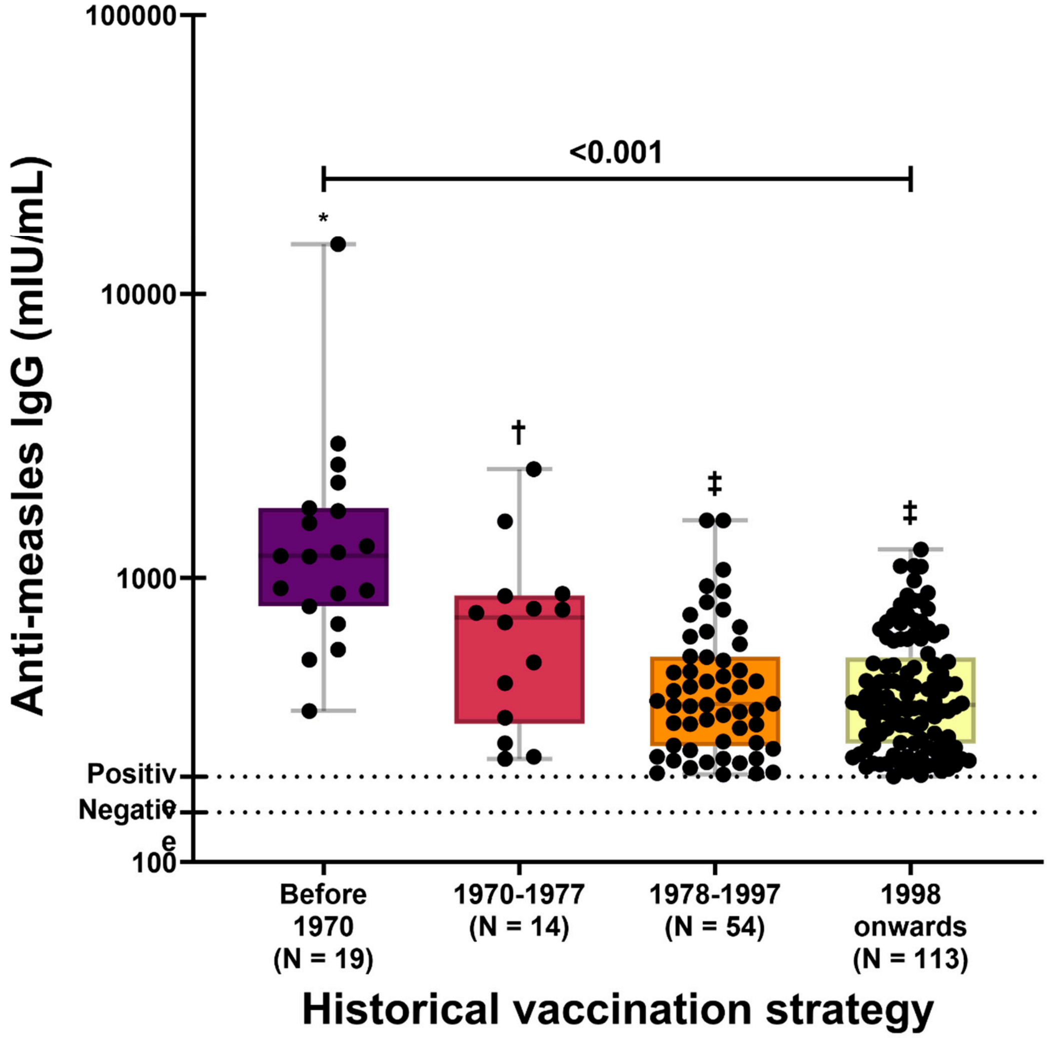

Supplementary Figure S3. Anti-measles IgG levels in seropositive individuals (>200 mIU/mL) according to historical measles vaccination strategies in Mexico. Data are presented on a logarithmic (log_{10}) scale. Group comparisons were performed using one-way ANOVA on log-transformed antibody concentrations, followed by Tukey's post hoc test. Different symbols above box plots indicate statistically significant differences between groups. The dotted horizontal lines denote the manufacturer's seropositivity threshold. The dotted horizontal lines denote the manufacturer's seropositivity threshold.
